# Online Tuning of PID Controller Using a Multilayer Fuzzy Neural Network Design for Quadcopter Attitude Tracking Control

**DOI:** 10.3389/fnbot.2020.619350

**Published:** 2021-01-18

**Authors:** Daewon Park, Tien-Loc Le, Nguyen Vu Quynh, Ngo Kim Long, Sung Kyung Hong

**Affiliations:** ^1^Faculty of Mechanical and Aerospace, Sejong University, Seoul, South Korea; ^2^Department of Convergence Engineering for Intelligent Drone, Sejong University, Seoul, South Korea; ^3^Faculty of Mechatronics and Electronics, Lac Hong University, Bien Hoa, Vietnam

**Keywords:** quadcopter attitude, fuzzy neural network, proportional-integral-derivative, attitude tracking control, fuzzy PID

## Abstract

This study presents an online tuning proportional-integral-derivative (PID) controller using a multilayer fuzzy neural network design for quadcopter attitude control. PID controllers are simple but effective control methods. However, finding the suitable gain of a model-based controller is relatively complicated and time-consuming because it depends on external disturbances and the dynamic modeling of plants. Therefore, the development of a method for online tuning of quadcopter PID parameters may save time and effort, and better control performance can be achieved. In our controller design, a multilayer structure was provided to improve the learning ability and flexibility of a fuzzy neural network. Adaptation laws to update network parameters online were derived using the gradient descent method. Also, a Lyapunov analysis was provided to guarantee system stability. Finally, simulations concerning quadcopter attitude control were performed using a Gazebo robotics simulator in addition to a robot operating system (ROS), and their results were demonstrated.

## Introduction

In the 4th industrial revolution era, the application of multi-copters has significantly expanded, and it has thus attracted the interest of many researchers specialized in multi-copter control engineering. Multi-copters need to maintain accurate attitudes to ensure stable flight, so the development of accurate and stable controllers for multi-copters is essential. The most popular controller used for multi-copters is the cascaded proportional-proportional integral derivative controller (PPID) (Hernandez and Frias, 2016; Salas et al., 2019; Santos et al., 2019; Xuan-Mung and Hong, 2019a,b). Since PID is a linear controller, it is generally hard to use to achieve the highest control performance for non-linear control systems (Sarabakha et al., 2017). Furthermore, in the design of a PID controller, it is necessary to obtain the exact mathematical model of the control system and to optimize the gain value to achieve the desired performance. However, this work requires complexity calculations and an accurate modeling plant.

Gain tuning for PID controllers is time-consuming, as it depends on the knowledge of dynamic plants and the experience of expert operators (Kim et al., 2007). Mathematical models are different, as they depend on the size and appearance of multi-copters. Even if it is the same aircraft, the center of gravity can be different (Kurak and Hodzic, 2018). Therefore, the gain values are always flexible, and it may take longer to find suitable values. However, such tasks must be completed correctly, as an incorrect calculation can lead to terrible problems, such as falling or bumping into a people or objects. In past decades, many studies have been conducted to solve such problems (time-consuming, costly, etc.; Fatan et al., 2013; Kuantama et al., 2017; Noordin et al., 2017; Rouhani et al., 2017; Sumardi and Riyadi, 2017; Prayitno et al., 2018; Thanh and Hong, 2018; Chen et al., 2019; Rabah et al., 2019; Soriano et al., 2020). The Ziegler Nichols method is a well-known for tuning PID parameters (Azman et al., 2017). However, it produces oscillatory responses and yields overshoot problems (Zahir et al., 2018). Moreover, it cannot be applied in the online-tuning of parameters, and the control performance can be further improved.

In recognizing the above-mentioned limitation of traditional PID controllers, many researchers have provided methods for auto-tuning PID parameters (Amoura et al., 2016; De Keyser et al., 2016; Concha et al., 2017; Live et al., 2017; Mendes et al., 2017; Wang, 2017; Bernardes et al., 2019). In which, using the fuzzy neural network to tune PID parameter controllers has attracted the attention of many researchers. In 2018, Davanipour et al. proposed a self-tuning PID controller based on a fuzzy wavelet neural network (Davanipour et al., 2018). In 2019, Tripathy et al. introduced a fuzzy PID controller for load frequency control using spider monkey optimization (Tripathy et al., 2019). In 2017, Wang et al. provided an improved fuzzy PID controller design using predictive functional control structure (Wang et al., 2017). These studies could reduce the effort required for tuning the required parameters for PID controllers. However, most of their methods were complicated, but the control performance could be further improved.

In recent years, many studies have investigated the fuzzy neural network (FNN), which is a combination of fuzzy systems and neural networks. Therefore, combined FNN systems have the advantages of both fuzzy systems and neural networks, such as human-like reasoning and learning capabilities. Using the neural network structure, FNN parameters can be trained online to achieve suitable values. According to the above discussions, this study presents a method for auto-tuning PID parameters using multilayer fuzzy neural network (PID-MFNN) to control the attitude the quadcopters. Compared with the previous studies (Wang et al., 2017; Davanipour et al., 2018; Tripathy et al., 2019), our proposed PID-MFNN has some advantages, such as no need for offline training. The major differences and improvements among this study and related works are shown in Table 1. Also, the fuzzy weights and fuzzy membership functions can be updated online, and the multilayer structure is applied to improve the control performance. The main contributions of this study are summarized as follows: (1) the successful design of a multilayer structure was provided to improve the learning ability and flexibility of a FNN; (2) the adaptation laws for updating the parameters of a controller online were derived by using the gradient descent method; (3) the simulation results of controlling the attitude of a quadcopter were provided to illustrate the effectiveness of the control design method.

**Table 1:** The major differences and improvements among this study and related works.

	**PID-MFNN (our proposed method)**	**FWNN-PID (Davanipour et al., 2018)**	**SMO-PID (Tripathy et al., 2019)**	**PFPID (Wang et al., 2017)**
Method	Using multilayer membership function neural network to online adjust the PID controller's parameters	Using fuzzy wavelet neural network to model the system for approximation the non-linear function. Then, the PID controller's parameters are designed based on the obtained model	Using the spider monkey optimization algorithm to optimize the PID controller's parameters	Using fuzzy system combined with PID to predictive functional model and control the process. The PID controller's parameters are tuned online by fuzzy inference
Membership function	Provided the adaptation laws for online updating the membership functions	Using fixed membership functions	Using fixed membership functions	Using predefined triangular membership functions and predefined sigmoid membership functions
Fuzzy weights	Provided the adaptation laws for online adjusting the fuzzy weights	Provided the adaptation laws for online adjusting the fuzzy weights	Using fixed fuzzy weights	Using predefined fuzzy rules based on technical knowledge and engineering design
Adaptation laws	Using the steepest descent gradient approach and a backpropagation algorithm	Using the steepest descent gradient approach and a backpropagation algorithm	Not use	Not use

The rest of this paper is organized as follows. The quadcopter dynamics model and quadcopter controller are given in section Problem Formulation. The PID-MFNN structure and its learning parameter are described in section PID-MFNN Structure and Parameter Learning. The simulation results of controlling the quadcopter attitude are presented in section Simulation Results. Finally, the conclusions are given in section Conclusion.

## Problem Formulation

### Quadcopter Dynamics Model

**Figure 1:**
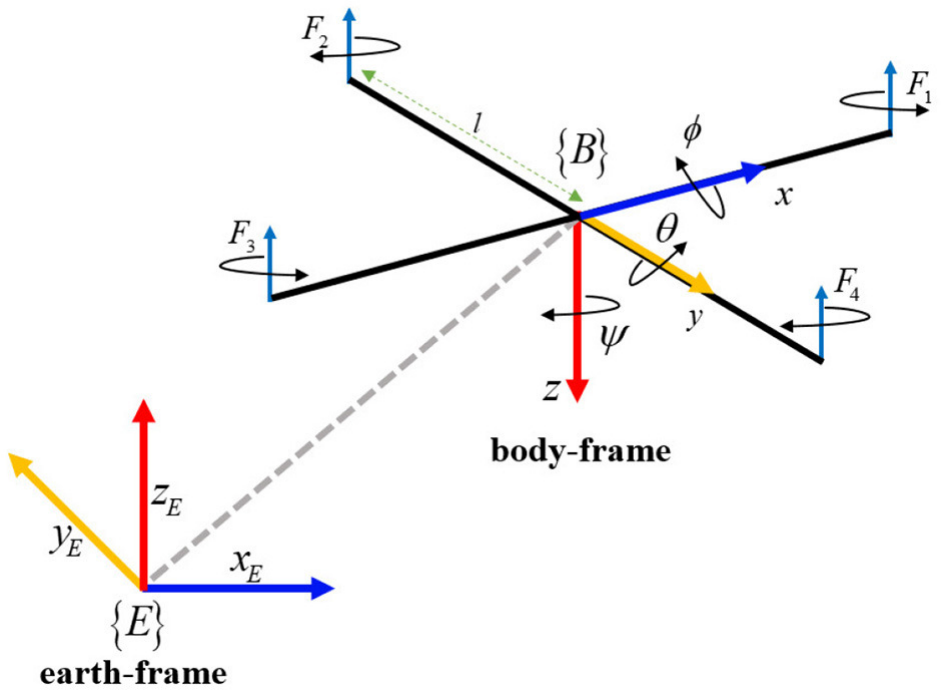
Quadcopter schematic and its reference frames.

The quadcopter schematic and its reference frames are shown in Figure 1. In which, the earth-frame and quadcopter body frame are denoted by *E*(*x*_*E*_, *y*_*E*_, *z*_*E*_) and *B*(*x, y, z*). The dynamic equations of the quadcopter are given as:

(1) $$\begin{array}{l} \{\begin{array}{c} ẍ=\frac{1}{M}\left(\cos\phi \sin\theta \cos\psi +\sin\phi \sin\psi \right)U_{1}\\ \ddot{y}=\frac{1}{M}\left(\cos\phi \sin\theta \sin\psi +\sin\phi \cos\psi \right)U_{1}\\ \ddot{z}=g-\frac{1}{M}\left(\cos\phi \cos\theta \right)U_{1}\\ \ddot{\phi }=\left(\frac{J_{y}-J_{z}}{J_{x}}\right)\dot{\theta }\dot{\psi }+\frac{l}{J_{x}}U_{2}\\ \ddot{\theta }=\left(\frac{J_{z}-J_{x}}{J_{y}}\right)\dot{\phi }\dot{\psi }+\frac{l}{J_{y}}U_{3}\\ \ddot{\psi }=\left(\frac{J_{x}-J_{y}}{J_{z}}\right)\dot{\phi }\dot{\theta }+\frac{1}{J_{z}}U_{4} \end{array} \end{array}$$

where θ, ϕ, and ψ stand for the three Euler angles: roll, pitch, and yaw, respectively; *J*_*x*_, *J*_*y*_ and *J*_*z*_ stand for the moment of inertia for the *x*, *y*, and *z* axes, respectively; *l* denotes the length of the quadcopter's arm; *U*_1_ denotes the total thrust on the body in the z-axis; *U*_2_, *U*_3_, and *U*_4_ denote the roll, pitch, and yaw torques, respectively, which are given as

(2) $$\begin{array}{l} \{\begin{array}{c} U_{1}=F_{1}+F_{3}+F_{2}+F_{4}\\ U_{2}=F_{4}-F_{2}\\ U_{3}=F_{3}-F_{1}\\ U_{4}=C_{d}\left(F_{1}+F_{3}-F_{2}-F_{4}\right) \end{array} \end{array}$$

where $F_{i}=C_{t}\Omega_{i}^{2}$ denotes the thrust of the *i*-th motor; Ω_*i*_ denotes the speed of the *i*-th motor; *C*_*t*_ and *C*_*d*_ stand for the thrust and drag coefficients, respectively.

### Quadcopter Controller

**Figure 2:**
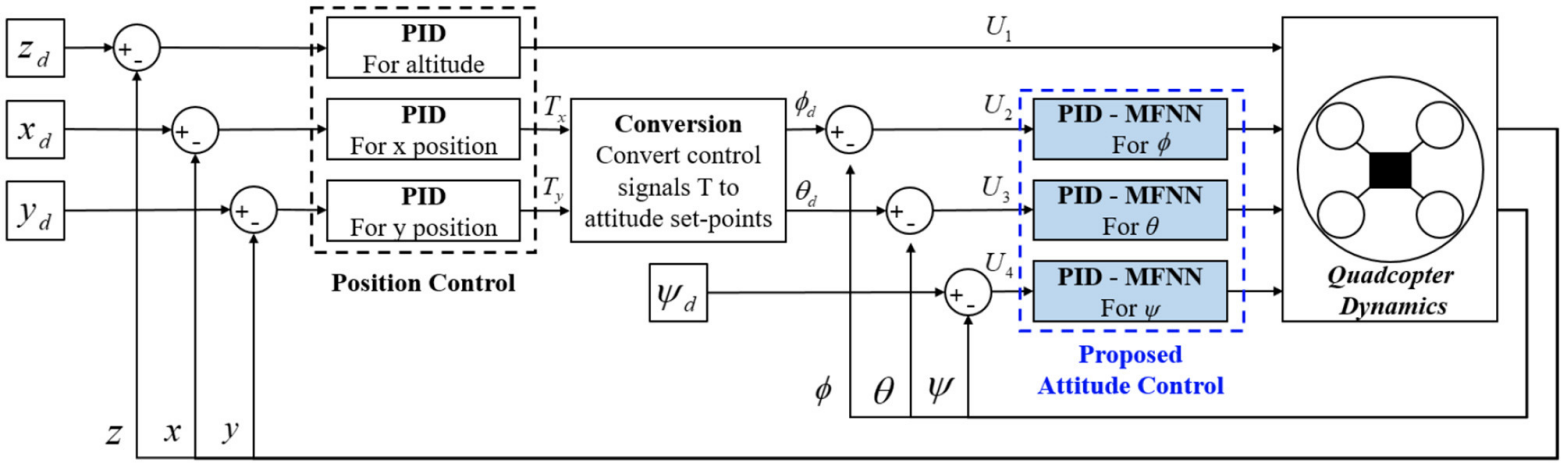
Quadcopter controller scheme.

**Figure 3:**
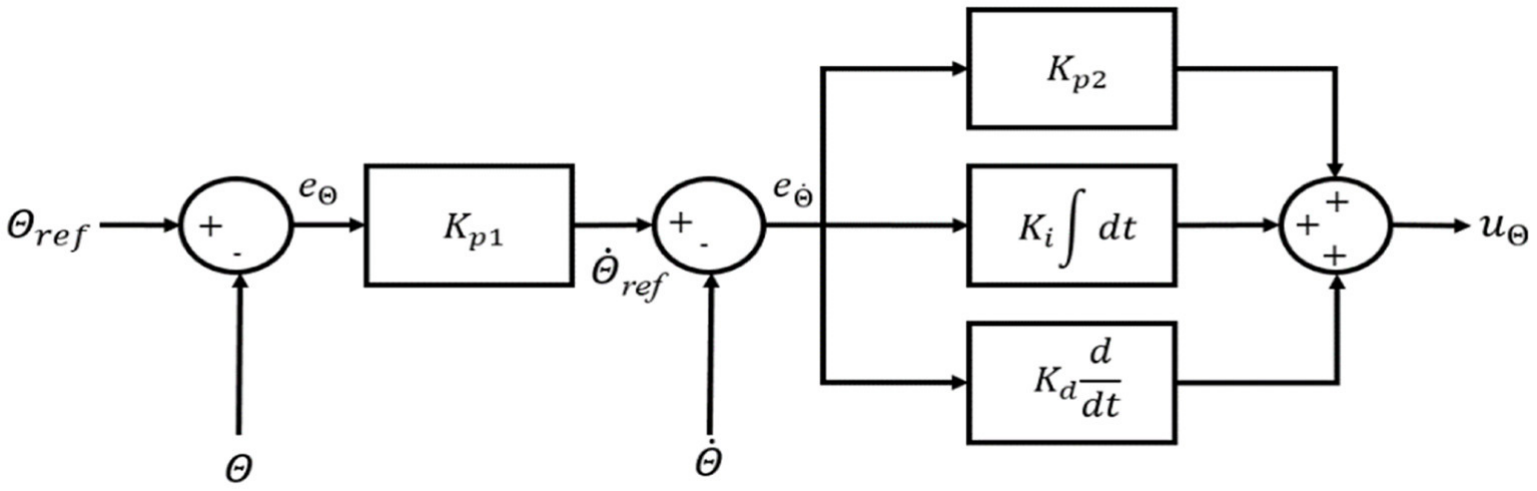
Control scheme of the quadcopter attitude controller (Θ is replaced for ϕ, θ, or ψ).

The quadcopter controller scheme is given in Figure 2. There are two kinds of controllers that need to be considered in quadcopter control: position controller and attitude controller. The PPID attitude controller is given in Figure 3. The output of the outer-loop controller is given by

(3) $$\begin{array}{l} \begin{array}{c} {\dot{\theta }}_{ref}=G_{\theta }e_{\theta }\\ {\dot{\phi }}_{ref}=G_{\phi }e_{\phi }\\ {\dot{\psi }}_{ref}=G_{\psi }e_{\psi } \end{array} \end{array}$$

where *G*_θ_, *G*_ϕ_, and *G*_ψ_ denote the proportional gain for the roll, pitch, and yaw outer-loop controller, respectively; *e*_θ_, *e*_ϕ_, and *e*_ψ_ are the angle errors of the roll, pitch, and yaw, respectively.

(4) $$\begin{array}{l} \begin{array}{c} e_{\theta }=\theta_{ref}-\theta \\ e_{\phi }=\phi_{ref}-\phi \\ e_{\psi }=\psi_{ref}-\psi \end{array} \end{array}$$

where θ_*ref*_, ϕ_*ref*_, and ψ_*ref*_ are the angle references of the roll, pitch, and yaw, respectively.

The output of the PID inner-loop controller is given by

(5) $$\begin{array}{l} \begin{array}{c} u_{\theta }=K_{p}^{\theta }e_{\dot{\theta }}+K_{i}^{\theta }\int_{0}^{t}e_{\dot{\theta }}+K_{d}^{\theta }{ė}_{\dot{\theta }}\\ u_{\phi }=K_{p}^{\phi }e_{\dot{\phi }}+K_{i}^{\phi }\int_{0}^{t}e_{\dot{\phi }}+K_{d}^{\phi }{ė}_{\dot{\phi }}\\ u_{\psi }=K_{p}^{\psi }e_{\dot{\psi }}+K_{i}^{\psi }\int_{0}^{t}e_{\dot{\psi }}+K_{d}^{\psi }{ė}_{\dot{\psi }} \end{array} \end{array}$$

where $K_{p}^{\theta }$, $K_{i}^{\theta }$, $K_{d}^{\theta }$, $K_{p}^{\phi }$, $K_{i}^{\phi }$, $K_{d}^{\phi }$, $K_{p}^{\psi }$, $K_{i}^{\psi }$, and $K_{d}^{\psi }$, are the PID gains for the roll, pitch, and yaw inner-loop controller; $e_{\dot{\theta }}$, $e_{\dot{\phi }}$, and $e_{\dot{\psi }}$ denote the angular velocity errors of the roll, pitch, and yaw, respectively.

(6) $$\begin{array}{l} \begin{array}{c} e_{\dot{\theta }}={\dot{\theta }}_{ref}-\dot{\theta }\\ e_{\dot{\phi }}={\dot{\phi }}_{ref}-\dot{\phi }\\ e_{\dot{\psi }}={\dot{\psi }}_{ref}-\dot{\psi } \end{array} \end{array}$$

To obtain suitable values for the PID gains in the outer-loop and inner-loop controller, the PID-MFNN was designed in the following section to adjust the gains for the PPID controller online.

## PID-MFNN Structure and Parameter Learning

### PID-MFNN Structure

The fuzzy rule forms for the proposed PID-MFNN controller are:

(7) $$\begin{array}{l} \begin{array}{c} l^{th}\text{rule}:IF\;i_{1}\;is\;\mu_{1jk}\;and\;i_{2}\;is\;\mu_{2jk},...,\;and\;i_{n_{i}}\;is\;\mu_{n_{i}jk}\\ Then\;out\;=\;w_{klo}\\ i=1,2,...,n_{i};j=1,2,...,n_{j};\\ k=1,2,...,n_{k};l=1,2,...,n_{l};o=1,2,...,n_{o} \end{array} \end{array}$$

where *i*_*i*_ is the fuzzy input; μ_*ijk*_ is the membership function (MF) for the input *i-th*, block *j-th*, and layer *k-th*; *w*_*klo*_ is the fuzzy weight for the layer *k-th*, rule *l-th*, and output *o-th*.

**Figure 4:**
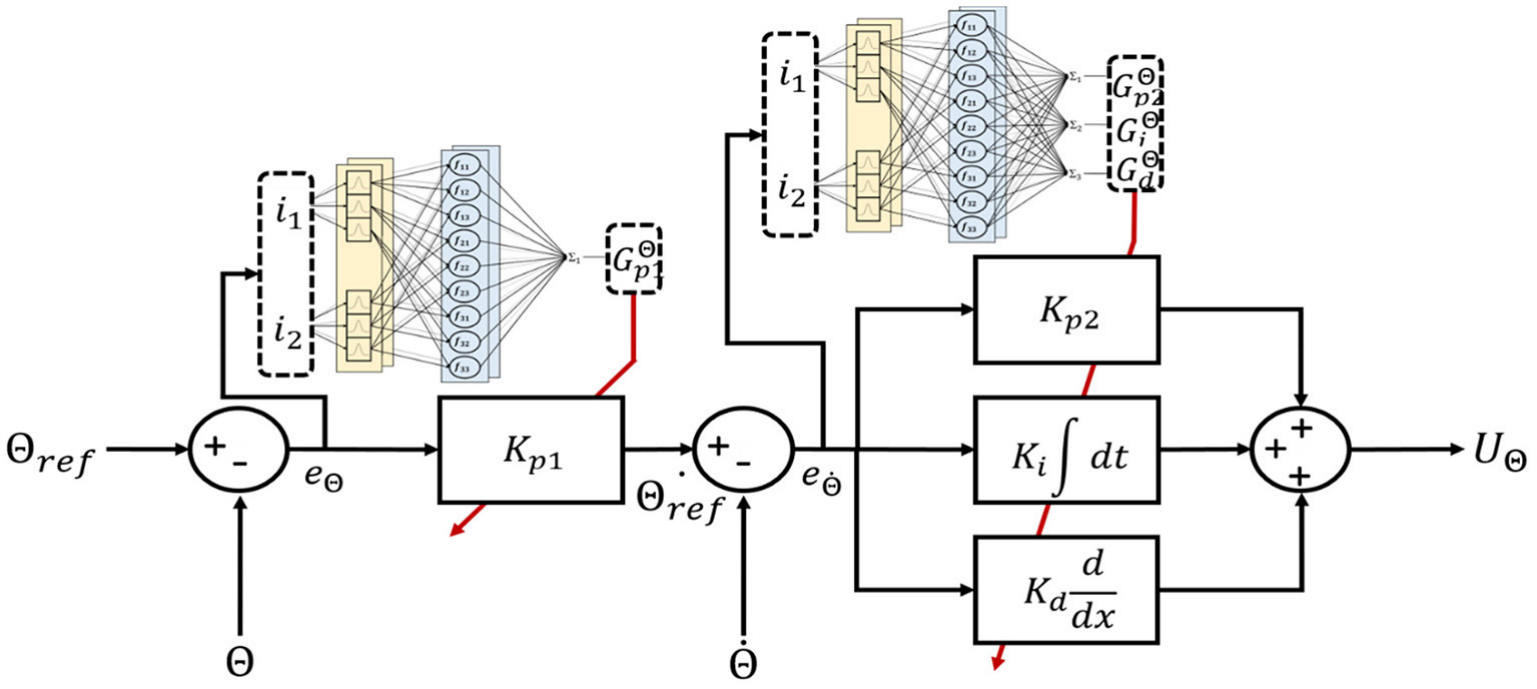
Auto-tuning algorithm structure of the PID-MFNN controller.

**Figure 5:**
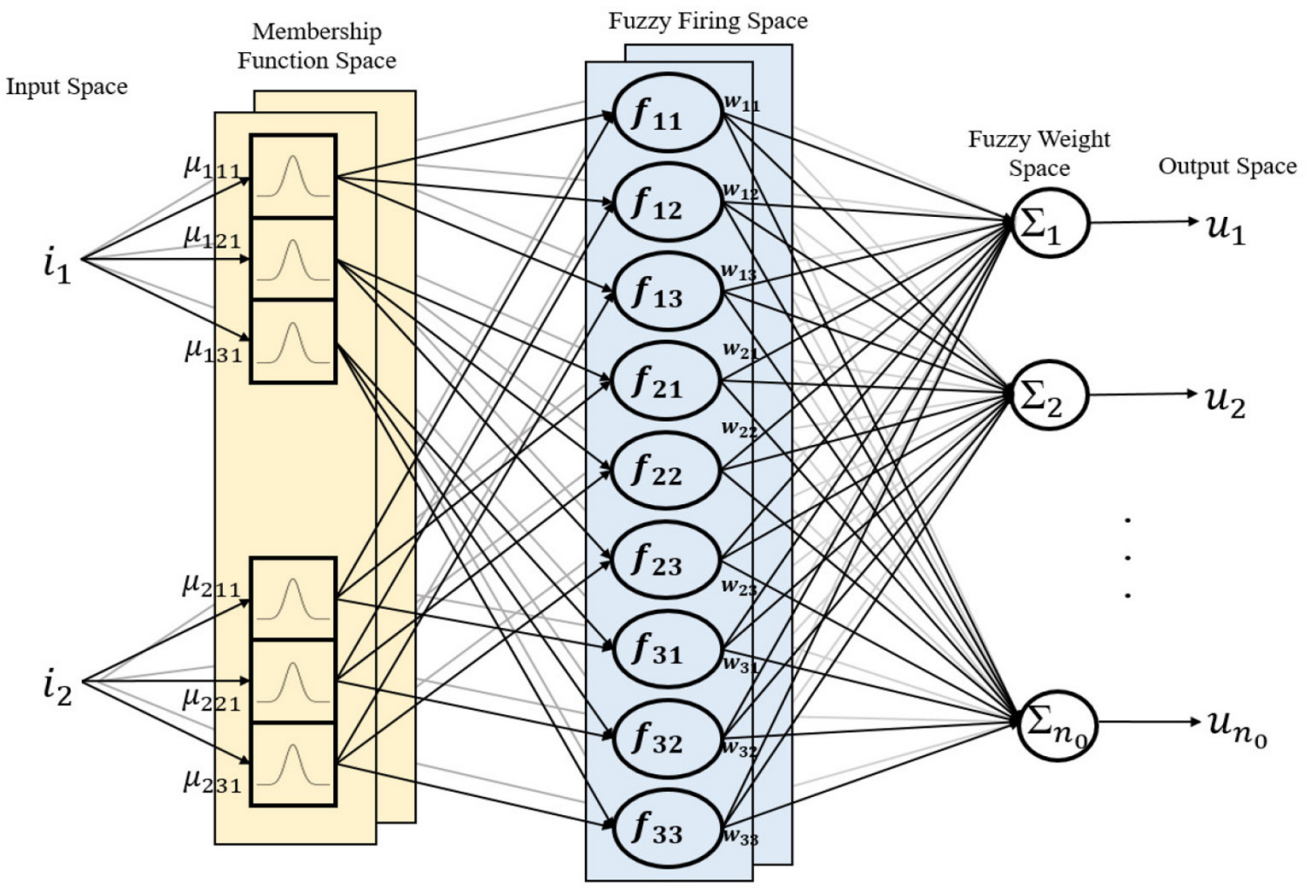
Multilayer fuzzy neural network structure.

**Figure 6:**
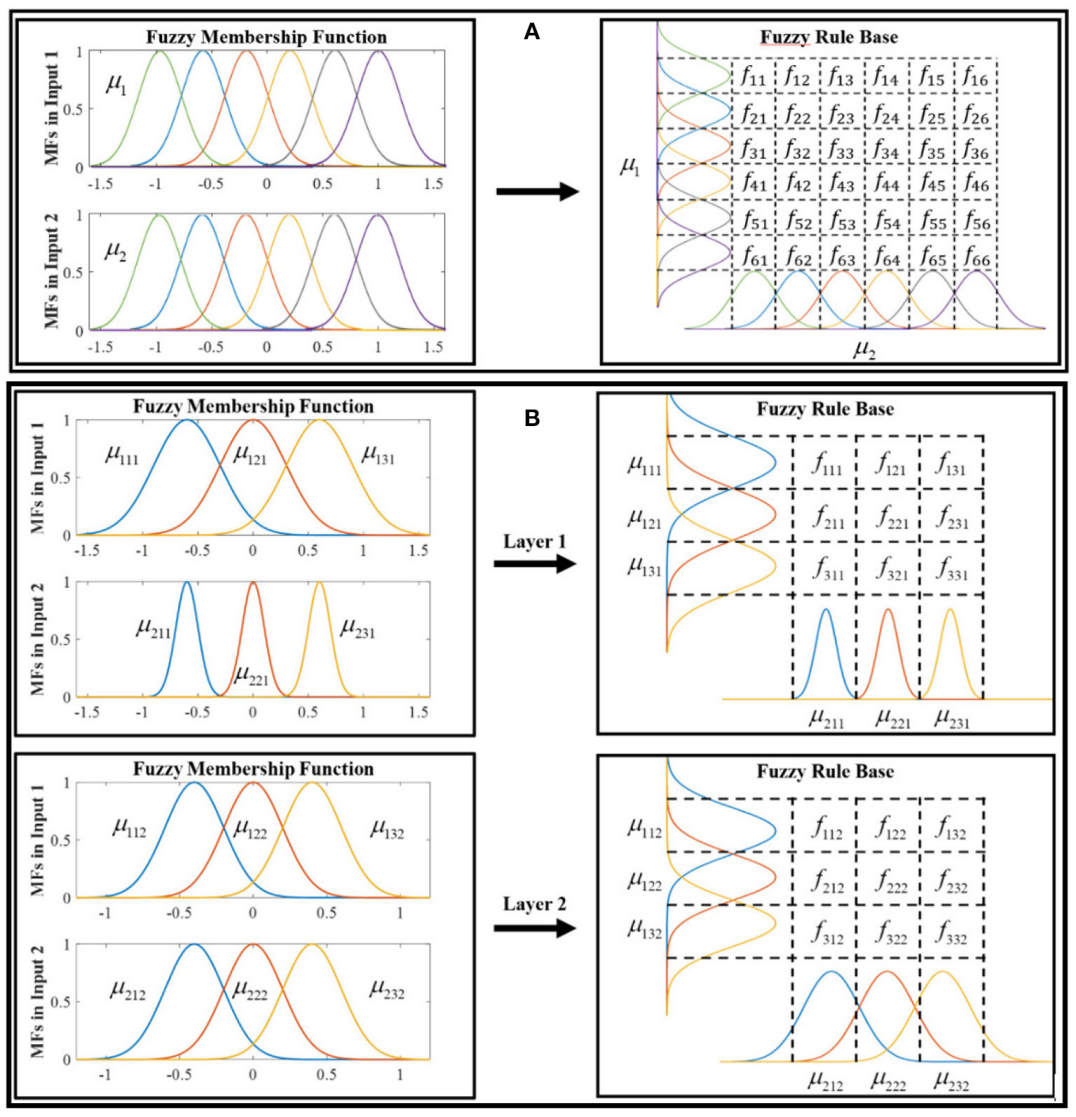
Fuzzy membership function and rule base: **(A)** the conventional fuzzy system and **(B)** the proposed multilayer fuzzy neural network with two layers.

The auto-tuning algorithm structure of the PID-MFNN controller is shown in Figure 4 in which, the MFNN structure is applied to adjust the PPID controller gains online. As shown in Figure 5, the MFNN structure includes five spaces as follows:

1. The input space: the fuzzy input vector, *I* = [*i*_1_, *i*_2_, …, *i*_*n*_*i*__], is prepared in this space. Each node corresponds to one input and is directly transferred to the next space.
2. The membership function space: the membership grades are calculated in this space using the fuzzy inputs and the Gaussian membership functions (GMFs). As shown in Figure 6, the multilayer fuzzy membership function is provided to improve the learning ability and flexibility of the FNN. The outputs of this space are computed as:

(8) $$\begin{array}{l} \mu_{ijk}=\text{exp}\left\{\frac{-{\left(i_{i}-m_{ijk}\right)}^{2}}{v_{ijk}^{2}}\right\} \end{array}$$

where *m*_*ijk*_ and *v*_*ijk*_ are the mean and variance of the GMFs.

3) The fuzzy firing space: the fuzzy firing strength is calculated in this space using the membership grades as follows:

(9) $$\begin{array}{l} f_{kl}=\overset{n_{i}}{\underset{i=1}{\prod }}\mu_{ijk} \end{array}$$

where *f*_*kl*_ is the fuzzy firing strength for the l-th rule in the k-th layer.

Then, the fuzzy firing vector is presented as

(10) $$\begin{array}{l} \left[\begin{array}{c} f_{1l}\\ f_{2l}\\ \vdots \\ f_{n_{k}l} \end{array}\right]=\left[\begin{array}{c} f_{11},f_{12},...,f_{1n_{l}}\\ f_{21},f_{22},...,f_{2n_{l}}\\ \vdots \vdots \vdots \\ f_{n_{k}1},f_{n_{k}2},...,f_{n_{k}n_{l}} \end{array}\right] \end{array}$$

4) The fuzzy weight space: in this space, the fuzzy weights *w*_*klo*_ are provided to connect the fuzzy firing space and output space. The fuzzy weight vector for the o-th output is presented as

(11) $$\begin{array}{l} \left[\begin{array}{c} w_{1lo}\\ w_{2lo}\\ \vdots \\ w_{n_{k}lo} \end{array}\right]=\left[\begin{array}{c} w_{11o},w_{12o},...,w_{1n_{l}o}\\ w_{21o},w_{22o},...,w_{2n_{l}o}\\ \vdots \vdots \vdots \\ w_{n_{k}1o},w_{n_{k}2o},...,w_{n_{k}n_{l}o} \end{array}\right] \end{array}$$

5) The output space: the final outputs of this space are the PID gains for the outer-loop and inner-loop controllers. Defuzzification is performed using the product operation as follows:

(12) $$\begin{array}{l} u_{o}=\overset{n_{k}}{\underset{k=1}{\sum }}\left(\frac{\overset{n_{l}}{\underset{l=1}{\sum }}\left(f_{kl}w_{klo}\right)}{\overset{n_{l}}{\underset{l=1}{\sum }}f_{kl}}\right) \end{array}$$

Using the proposed MFNN to adjust the PID gains online, the desired performance of the control network can be achieved. The adaptation laws for updating the parameters of the proposed PID-MFNN controller online are explained in the following section.

### Parameter Learning

First, considering the outer-loop controller, Equation (3) can be rewritten, where the gains *G*_θ_, *G*_ϕ_, and *G*_ψ_ are obtained using the output of the proposed MFNN in Equation (10).

(13) $$\begin{array}{l} {\dot{\Theta }}_{ref}=G_{\Theta }e_{\Theta } \end{array}$$

(14) $$\begin{array}{l} G_{\Theta }=\overset{n_{k}}{\underset{k=1}{\sum }}\left(\frac{\overset{n_{l}}{\underset{l=1}{\sum }}\left(f_{kl}w_{kl\Theta }^{g}\right)}{\overset{n_{l}}{\underset{l=1}{\sum }}f_{kl}}\right) \end{array}$$

where Θ is replaced by (θ, ϕ, ψ).

By rewriting the Equation (6),

(15) $$\begin{array}{l} e_{\dot{\Theta }}={\dot{\Theta }}_{ref}-\dot{\Theta } \end{array}$$

A Lyapunov function is defined as $\;E_{1}\left(t\right)=\frac{1}{2}{\left(e_{\dot{\Theta }}\left(t\right)\right)}^{2}$, using the gradient descent approach and chain rule, and the parameter update laws for PID-MFNN can be obtained as follows:

(16) $$\begin{array}{l} {ŵ}_{kl\Theta }^{g}\left(t+1\right)={ŵ}_{kl\Theta }^{g}\left(t\right)-\eta_{w}^{g}\frac{\partial E_{1}}{\partial {ŵ}_{kl\Theta }^{g}}\\ \;={ŵ}_{kl\Theta }^{g}\left(t\right)-\eta_{w}^{g}\frac{\partial E_{1}}{\partial e_{\dot{\Theta }}}\frac{\partial e_{\dot{\Theta }}}{\partial {\dot{\Theta }}_{ref}}\frac{\partial {\dot{\Theta }}_{ref}}{\partial G_{\Theta }}\frac{\partial G_{\Theta }}{\partial {ŵ}_{kl\Theta }^{g}}\\ \;={ŵ}_{kl\Theta }^{g}\left(t\right)-\eta_{w}^{g}e_{\dot{\Theta }}e_{\Theta }\frac{f_{kl}}{\overset{n_{l}}{\underset{l=1}{\sum }}f_{kl}} \end{array}$$

(17) $$\begin{array}{l} {\hat{m}}_{ijk}^{g}\left(t+1\right)={\hat{m}}_{ijk}^{g}\left(t\right)-\eta_{m}^{g}\frac{\partial E_{1}}{\partial {\hat{m}}_{ijk}^{g}}\\ \;={\hat{m}}_{ijk}^{g}\left(t\right)-\eta_{m}^{g}\frac{\partial E_{1}}{\partial e_{\dot{\Theta }}}\frac{\partial e_{\dot{\Theta }}}{\partial {\dot{\Theta }}_{ref}}\frac{\partial {\dot{\Theta }}_{ref}}{\partial G_{\Theta }}\frac{\partial G_{\Theta }}{\partial f_{kl}}\frac{\partial f_{kl}}{\partial \mu_{ijk}}\frac{\partial \mu_{ijk}}{\partial {\hat{m}}_{ijk}^{g}}\\ \;={\hat{m}}_{ijk}^{g}\left(t\right)-\eta_{m}^{g}e_{\dot{\Theta }}e_{\Theta }\frac{\left({ŵ}_{kl\Theta }^{g}-G_{\Theta }\right)}{\overset{n_{l}}{\underset{l=1}{\sum }}f_{kl}}f_{kl}\frac{\left(i_{i}-{\hat{m}}_{ijk}^{g}\right)}{{\left({\hat{v}}_{ijk}^{g}\right)}^{2}} \end{array}$$

(18) $$\begin{array}{l} {\hat{v}}_{ijk}^{g}\left(t+1\right)={\hat{v}}_{ijk}^{g}\left(t\right)-\eta_{v}^{g}\frac{\partial E_{1}}{\partial {\hat{v}}_{ijk}^{g}}\\ \;={\hat{v}}_{ijk}^{g}\left(t\right)-\eta_{v}^{g}\frac{\partial E_{1}}{\partial e_{\dot{\Theta }}}\frac{\partial e_{\dot{\Theta }}}{\partial {\dot{\Theta }}_{ref}}\frac{\partial {\dot{\Theta }}_{ref}}{\partial G_{\Theta }}\frac{\partial G_{\Theta }}{\partial f_{kl}}\frac{\partial f_{kl}}{\partial \mu_{ijk}}\frac{\partial \mu_{ijk}}{\partial {\hat{v}}_{ijk}^{g}}\\ \;={\hat{v}}_{ijk}^{g}\left(t\right)-\eta_{v}^{g}e_{\dot{\Theta }}e_{\Theta }\frac{\left({ŵ}_{kl\Theta }^{g}-G_{\Theta }\right)}{\overset{n_{l}}{\underset{l=1}{\sum }}f_{kl}}f_{kl}\frac{{\left(i_{i}-{\hat{m}}_{ijk}^{g}\right)}^{2}}{{\left({\hat{v}}_{ijk}^{g}\right)}^{3}} \end{array}$$

where ${ŵ}_{kl\Theta }^{g}$, ${\hat{m}}_{ijk}^{g}$, and ${\hat{v}}_{ijk}^{g}$ are the connecting weight, mean, and variance of the outer-loop MFNN controller; $\eta_{w}^{g}$, $\eta_{m}^{g}$, and $\eta_{v}^{g}$ are the positive learning rates.

Then, considering the inner-loop controller, Equation (5) can be rewritten, where the gains $K_{p}^{\theta }$, $K_{i}^{\theta }$, $K_{d}^{\theta }$, $K_{p}^{\phi }$, $K_{i}^{\phi }$, $K_{d}^{\phi }$, $K_{p}^{\psi }$, $K_{i}^{\psi }$, and $K_{d}^{\psi }$ are obtained using the outputs of the proposed MFNN.

(19) $$\begin{array}{l} u^{\Theta }=u_{p}^{\Theta }+u_{i}^{\Theta }+u_{d}^{\Theta }=K_{p}^{\Theta }G_{p}^{\Theta }+K_{i}^{\Theta }G_{i}^{\Theta }+K_{d}^{\Theta }G_{d}^{\Theta } \end{array}$$

where $G_{p}^{\Theta }=e_{\dot{\Theta }}$; $G_{i}^{\Theta }=\overset{t}{\underset{0}{\int }}e_{\dot{\Theta }}$; $G_{d}^{\Theta }={ė}_{\dot{\Theta }}$

(20) $$\begin{array}{l} K_{p}^{\Theta }=\overset{n_{k}}{\underset{k=1}{\sum }}\left(\frac{\overset{n_{l}}{\underset{l=1}{\sum }}\left(f_{kl}w_{kl\Theta }^{p}\right)}{\overset{n_{l}}{\underset{l=1}{\sum }}f_{kl}}\right);K_{i}^{\Theta }=\overset{n_{k}}{\underset{k=1}{\sum }}\left(\frac{\overset{n_{l}}{\underset{l=1}{\sum }}\left(f_{kl}w_{kl\Theta }^{i}\right)}{\overset{n_{l}}{\underset{l=1}{\sum }}f_{kl}}\right);\\ K_{d}^{\Theta }=\overset{n_{k}}{\underset{k=1}{\sum }}\left(\frac{\overset{n_{l}}{\underset{l=1}{\sum }}\left(f_{kl}w_{kl\Theta }^{d}\right)}{\overset{n_{l}}{\underset{l=1}{\sum }}f_{kl}}\right) \end{array}$$

Then

(21) $$\begin{array}{l} K_{\alpha }^{\Theta }=\overset{n_{k}}{\underset{k=1}{\sum }}\left(\frac{\overset{n_{l}}{\underset{l=1}{\sum }}\left(f_{kl}w_{kl\Theta }^{\alpha }\right)}{\overset{n_{l}}{\underset{l=1}{\sum }}f_{kl}}\right);u_{\alpha }^{\Theta }=K_{\alpha }^{\Theta }G_{\alpha }^{\Theta } \end{array}$$

where α is replaced by (*p, i, d*).

By rewriting the Equation (4),

(22) $$\begin{array}{l} e_{\Theta }=\Theta_{ref}-\Theta \end{array}$$

A Lyapunov function can be defined as $\;E_{2}\left(t\right)=\frac{1}{2}{\left(e_{\Theta }\left(t\right)\right)}^{2}$, using the gradient descent approach and chain rule, and the parameter update laws for PID-MFNN can be obtained as:

(23) $$\begin{array}{l} {ŵ}_{kl\Theta }^{\alpha }\left(t+1\right)={ŵ}_{kl\Theta }^{\alpha }\left(t\right)-\eta_{w}^{\alpha }\frac{\partial E_{2}}{\partial {ŵ}_{kl\Theta }^{\alpha }}\\ \;={ŵ}_{kl\Theta }^{\alpha }\left(t\right)-\eta_{w}^{\alpha }\frac{\partial E_{2}}{\partial e_{\Theta }}\frac{\partial e_{\Theta }}{\partial \Theta }\frac{\partial \Theta }{\partial u_{\alpha }^{\Theta }}\frac{\partial u_{\alpha }^{\Theta }}{\partial K_{\alpha }^{\Theta }}\frac{\partial K_{\alpha }^{\Theta }}{\partial {ŵ}_{kl\Theta }^{\alpha }}\\ \;={ŵ}_{kl\Theta }^{\alpha }\left(t\right)+\eta_{w}^{\alpha }e_{\Theta }T_{\Theta }G_{\alpha }^{\Theta }\frac{f_{kl}}{\overset{n_{l}}{\underset{l=1}{\sum }}f_{kl}} \end{array}$$

(24) $$\begin{array}{l} {\hat{m}}_{ijk}^{\alpha }\left(t+1\right)={\hat{m}}_{ijk}^{\alpha }\left(t\right)-\eta_{m}^{\alpha }\frac{\partial E_{2}}{\partial {\hat{m}}_{ijk}^{\alpha }}\\ \;={\hat{m}}_{ijk}^{\alpha }\left(t\right)-\eta_{m}^{\alpha }\frac{\partial E_{2}}{\partial e_{\Theta }}\frac{\partial e_{\Theta }}{\partial \Theta }\frac{\partial \Theta }{\partial u_{\alpha }^{\Theta }}\frac{\partial u_{\alpha }^{\Theta }}{\partial K_{\alpha }^{\Theta }}\frac{\partial K_{\alpha }^{\Theta }}{\partial f_{kl}}\frac{\partial f_{kl}}{\partial \mu_{ijk}}\frac{\partial \mu_{ijk}}{\partial {\hat{m}}_{ijk}^{\alpha }}\\ \;={\hat{m}}_{ijk}^{\alpha }\left(t\right)+\eta_{m}^{\alpha }e_{\Theta }T_{\Theta }G_{\alpha }^{\Theta }\frac{\left({ŵ}_{kl\Theta }^{\alpha }-K_{\alpha }^{\Theta }\right)}{\overset{n_{l}}{\underset{l=1}{\sum }}f_{kl}}f_{kl}\frac{\left(i_{i}-{\hat{m}}_{ijk}^{\alpha }\right)}{{\left({\hat{v}}_{ijk}^{\alpha }\right)}^{2}} \end{array}$$

(25) $$\begin{array}{l} {\hat{v}}_{ijk}^{\alpha }\left(t+1\right)={\hat{v}}_{ijk}^{\alpha }\left(t\right)-\eta_{v}^{\alpha }\frac{\partial E_{2}}{\partial {\hat{v}}_{ijk}^{\alpha }}\\ \;={\hat{v}}_{ijk}^{\alpha }\left(t\right)-\eta_{v}^{\alpha }\frac{\partial E_{2}}{\partial e_{\Theta }}\frac{\partial e_{\Theta }}{\partial \Theta }\frac{\partial \Theta }{\partial u_{\alpha }^{\Theta }}\frac{\partial u_{\alpha }^{\Theta }}{\partial K_{\alpha }^{\Theta }}\frac{\partial K_{\alpha }^{\Theta }}{\partial f_{kl}}\frac{\partial f_{kl}}{\partial \mu_{ijk}}\frac{\partial \mu_{ijk}}{\partial {\hat{v}}_{ijk}^{\alpha }}\\ \;={\hat{v}}_{ijk}^{\alpha }\left(t\right)+\eta_{v}^{\alpha }e_{\Theta }T_{\Theta }G_{\alpha }^{\Theta }\frac{\left({ŵ}_{kl\Theta }^{\alpha }-K_{\alpha }^{\Theta }\right)}{\overset{n_{l}}{\underset{l=1}{\sum }}f_{kl}}f_{kl}\frac{{\left(i_{i}-{\hat{m}}_{ijk}^{\alpha }\right)}^{2}}{{\left({\hat{v}}_{ijk}^{\alpha }\right)}^{3}} \end{array}$$

where ${ŵ}_{kl\Theta }^{\alpha }$, ${\hat{m}}_{ijk}^{\alpha }$, and ${\hat{v}}_{ijk}^{\alpha }$ are the connecting weight, mean, and variance of the inner-loop MFNN controller; *T*_Θ_ can be *T*_ϕ_, *T*_θ_, or *T*_Ψ_, which are the quadcopter transfer functions for the yaw, pitch and roll angles; $\eta_{w}^{\alpha }$, $\eta_{m}^{\alpha }$, and $\eta_{v}^{\alpha }$ are the positive learning rates.

Using the proposed adaptation laws in (16–18) and (23–25), the parameters of the proposed PID-MFNN can be obtained. Then, the suitable gains for the outer-loop and inner-loop PPID controller can be achieved.

#### The Convergence Analysis

The Lyapunov cost function is defined as

(26) $$\begin{array}{l} V\left(t\right)=E_{1}=\frac{1}{2}{\left(e_{\dot{\Theta }}\left(t\right)\right)}^{2} \end{array}$$

Then,

(27) $$\begin{array}{l} \Delta V\left(t\right)=V\left(t+1\right)-V\left(t\right)=\frac{1}{2}\left[{\left(e_{\dot{\Theta }}\left(t+1\right)\right)}^{2}-{\left(e_{\dot{\Theta }}\left(t\right)\right)}^{2}\right] \end{array}$$

By applying the Taylor expansion,

(28) $$\begin{array}{l} e_{\dot{\Theta }}\left(t+1\right)=e_{\dot{\Theta }}\left(t\right)+\Delta e_{\dot{\Theta }}\left(t\right)≅e_{\dot{\Theta }}\left(t\right)+\left[\frac{\partial e_{\dot{\Theta }}\left(t\right)}{\partial {ŵ}_{kl\Theta }^{g}}\right]\Delta {ŵ}_{kl\Theta }^{g} \end{array}$$

From (16),

(29) $$\begin{array}{l} \frac{\partial e_{\dot{\Theta }}\left(t\right)}{\partial {ŵ}_{kl\Theta }^{g}}=e_{\Theta }\frac{f_{kl}}{\overset{n_{l}}{\underset{l=1}{\sum }}f_{kl}}=\zeta \end{array}$$

By rewriting (28) using (29) and (16),

(30) $$\begin{array}{l} e_{\dot{\Theta }}\left(t+1\right)=e_{\dot{\Theta }}\left(t\right)-\zeta \left(\eta_{w}^{g}e_{\dot{\Theta }}\left(t\right)\zeta \right)=e_{\dot{\Theta }}\left(t\right)\left[1-\eta_{w}^{g}\zeta^{2}\right] \end{array}$$

By rewriting (27) using (30),

(31) $$\begin{array}{l} \Delta V\left(t\right)=\frac{1}{2}{\left(e_{\dot{\Theta }}\left(t\right)\right)}^{2}\left[{\left(1-\eta_{w}^{g}\zeta^{2}\right)}^{2}-1\right]\\ \;=\frac{1}{2}{\left(e_{\dot{\Theta }}\left(t\right)\right)}^{2}\left[{\left(\eta_{w}^{g}\zeta^{2}\right)}^{2}-2\eta_{w}^{g}\zeta^{2}\right]\\ \;=\frac{1}{2}\eta_{w}^{g}{\left(e_{\dot{\Theta }}\left(t\right)\right)}^{2}\zeta^{2}\left(\eta_{w}^{g}\zeta^{2}-2\right) \end{array}$$

From (31), if $\eta_{w}^{g}$ is chosen to satisfy $0<\eta_{w}^{g}<\frac{2}{\zeta^{2}}$, then Δ*V*(*t*) < 0. Consequently, the convergence of the adaptation law is guaranteed by the Lyapunov theorem. Similarly, the convergence of the adaptation laws for $\eta_{w}^{\alpha }$, $\eta_{m}^{\alpha }$, and $\eta_{v}^{\alpha }$ can be obtained.

The computational complexity of our approaches using big O notation is given as:

+ Big O notation for PID-CFNN:

(32) $$\begin{array}{l} Big-O=O\left(T*\left[\max\left(n_{1},n_{2}\right)+n_{1}*n_{2}\right]\right) \end{array}$$

+ Big O notation for PID-MFNN:

(33) $$\begin{array}{l} Big-O=O\left(T*n_{k}\left[\max\left(n_{j_{1}},n_{j_{2}}\right)+\left(n_{j_{1}}*n_{j_{2}}\right)\right]\right) \end{array}$$

where:

T: is the simulation time.
*n*_*j*_1__: the number of membership functions in input 1.
*n*_*j*_2__: the number of membership functions in input 2.
*n*_*k*_: the number of layers.

In our proposed network, we choose *n*_*j*_1__ = *n*_*j*_2__ = 3 and *n*_*k*_ = 2.

## Simulation Results

This section presents the results of the quadcopter attitude control using the proposed PID-MFNN controller, which is performed using the Gazebo robotics simulator and ROS. The Gazebo simulation is a multi-robot simulator for outdoor environments. It supports the interface of the quadcopter's firmware, Pixhawk 4 (PX4). Therefore, it is easy to implement our algorithm on the quadcopter and to receive or transmit necessary data. The basic commands using MAVlink are also supported. Inside PX4, many terrains, sensors, and quadcopter types are provided using JavaScript. In this study, the 3D Robotics IRIS quadcopter was used without additional sensors, which were simulated on the basic terrain as a real world environment for verifying our designed control system. The wind disturbances of 1.0 m/s are considered as external disturbances.

The quadcopter parameters used in this model were chosen as shown in Table 2.

**Table 2:** The 3DR's IRIS parameters inside the Gazebo simulation used in the experiment.

**Parameter**	**Value**	**Unit**
*m*	1.5	*kg*
*l*	0.275	*m*
*h*	0.11	*m*
*g*	9.81	*m/s*^2^
*J*_*x*_	0.0347563	*kg*·*m*^2^
*J*_*y*_	0.0458929	*kg*·*m*^2^
*J*_*z*_	0.0977	*kg*·*m*^2^
*C*_*t*_	8.54858e-06	*N*·*s*^2^
*C*_*d*_	0.000806428	*N*·*m*·*s*^2^

The goal of the control system is to control the attitude of the quadcopter following the reference set-point trajectory:

(34) $$\begin{array}{l} \theta_{ref}=5*square\left(0.2\pi t\right)\\ \phi_{ref}=5*square\left(0.2\pi t\right)\\ \psi_{ref}=90+5*square\left(0.2\pi t\right) \end{array}$$

(35) $$\begin{array}{l} \theta_{ref}=5*sin\left(0.2\pi t\right)\\ \phi_{ref}=5*sin\left(0.2\pi t\right)\\ \psi_{ref}=90+5*sin\left(0.2\pi t\right) \end{array}$$

**Figure 7:**
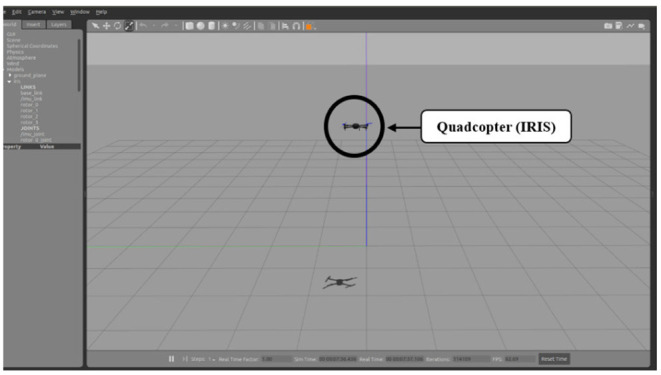
Gazebo simulation interface.

**Figure 8:**
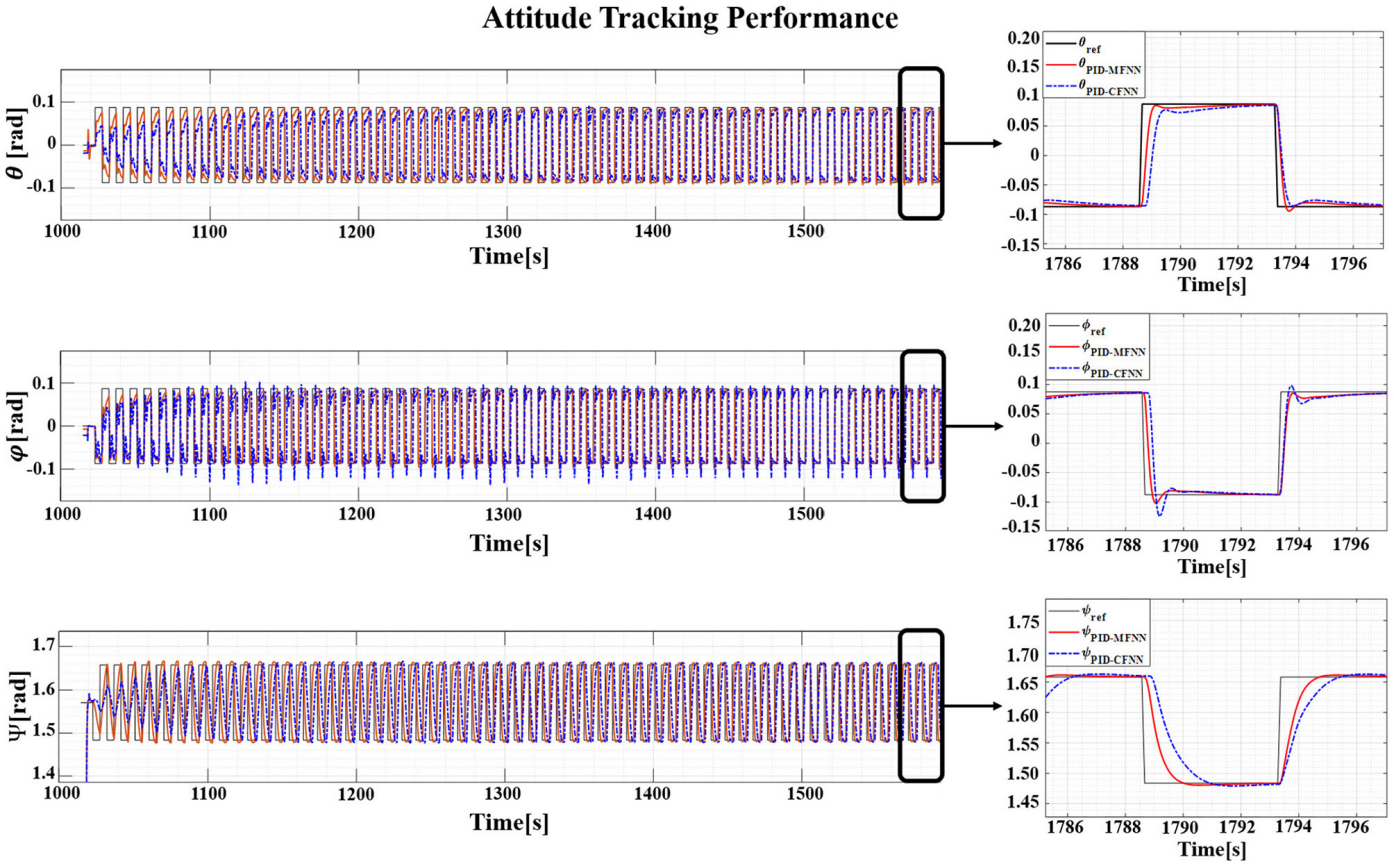
The tracking performance for the quadcopter attitude control for the square wave commands.

**Figure 9:**
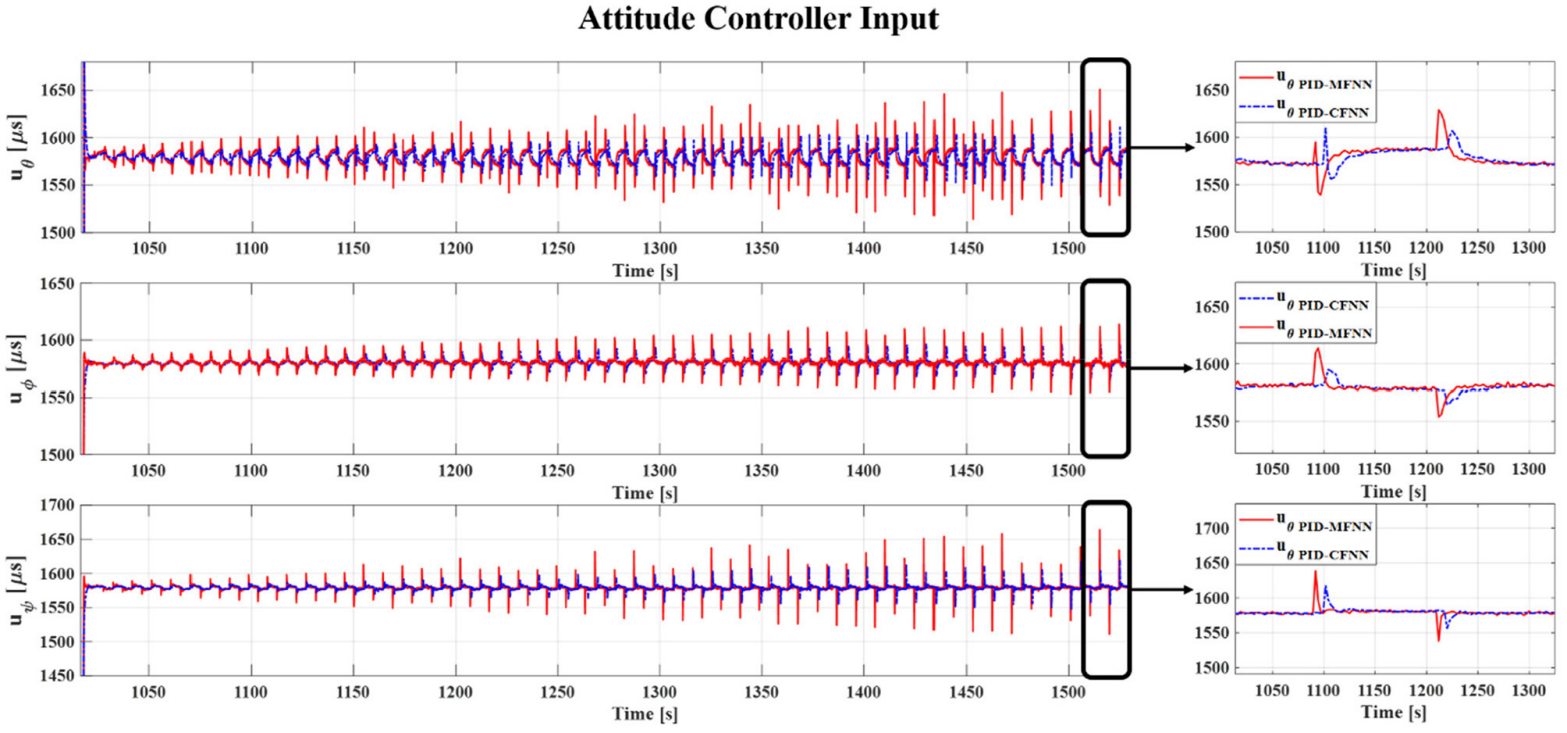
The control signals for the quadcopter attitude control for the square wave commands.

**Figure 10:**
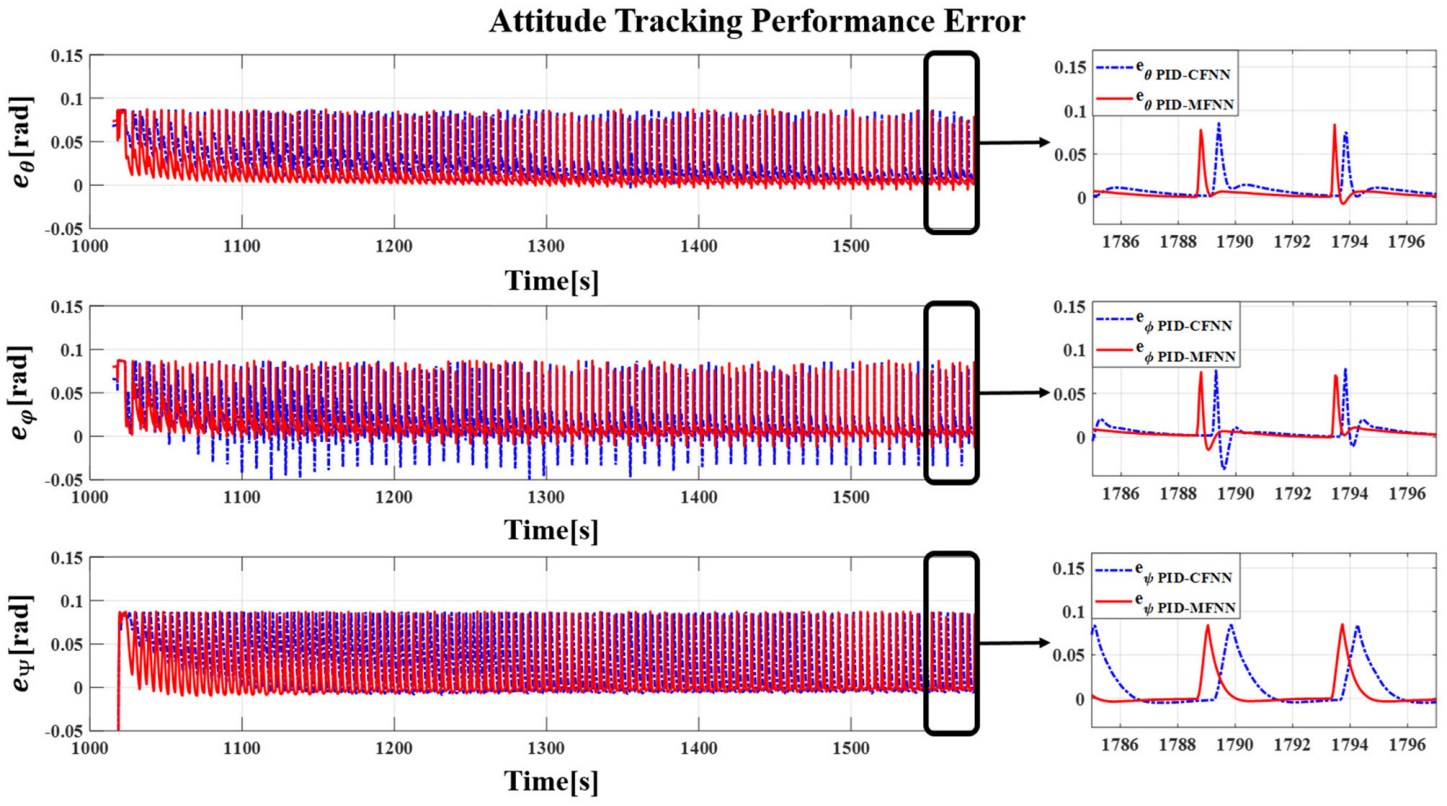
The tracking errors for the quadcopter attitude control for the square wave commands.

**Figure 11:**
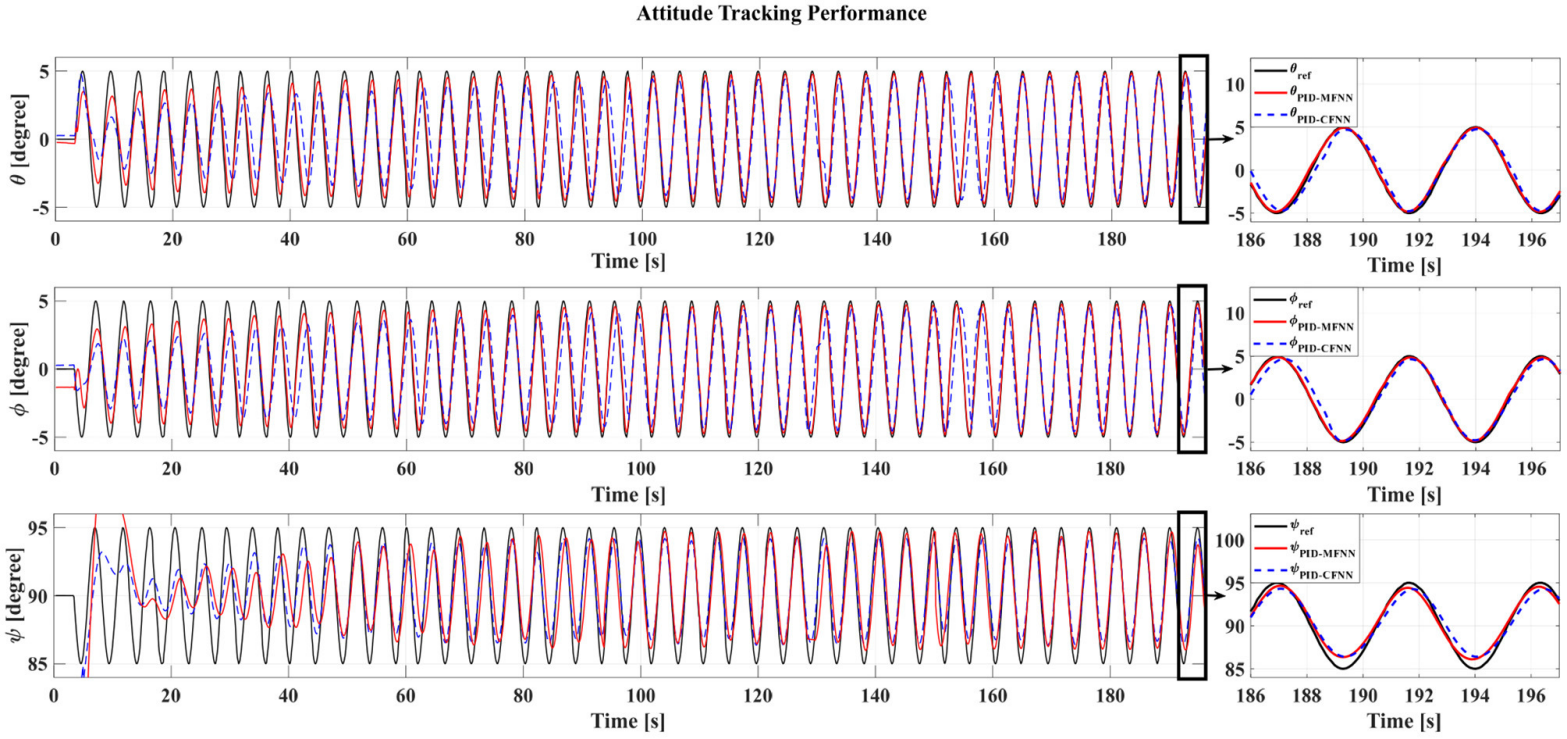
The quadcopter attitude control tracking performance for the sine wave commands.

**Figure 12:**
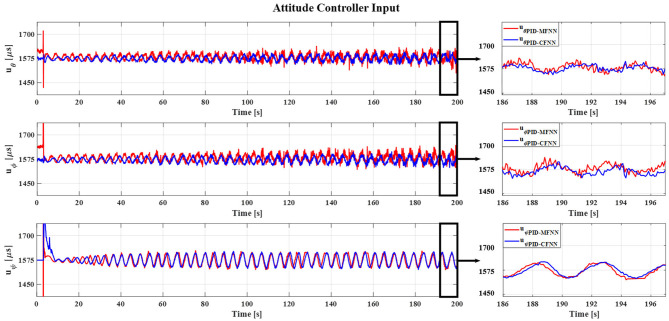
The quadcopter attitude controller input for the sine wave commands.

**Figure 13:**
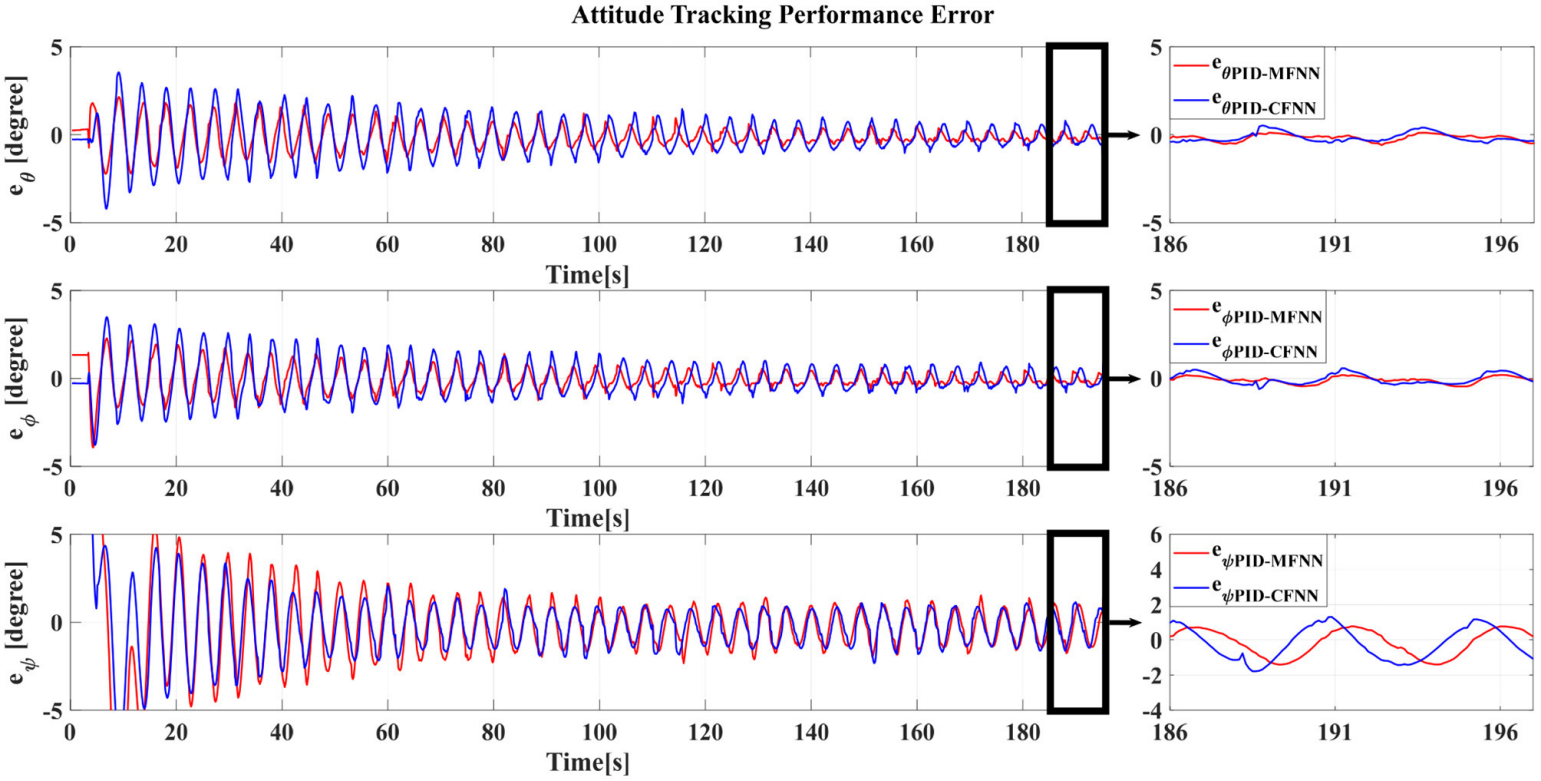
The quadcopter attitude control tracking performance error for the sine wave commands.

The Gazebo simulation interface is shown in Figure 7. The tracking performances of the quadcopter attitude under square wave and sine wave commands are given in Figures 8, 11, respectively. The black line presents the reference set-point trajectory. The red and blue lines present the quadcopter attitude output using the PID-MFNN controller and the PID conventional fuzzy controller (PID-CFNN), respectively. The control signals under the square wave and sine wave commands are shown in Figures 9, 12, respectively. The tracking errors under the square wave and sine wave commands are given in Figures 10, 13, respectively. The comparison results concerning the root mean square error (RMSE) between the proposed PID-MFNN controller and PID-CFNN controller are given in Tables 3, 4. From Figures 8–13, it can be seen that the proposed PID-MFNN controller has better control performance with a faster convergence speed and smaller tracking errors compared with the conventional fuzzy controller, which demonstrates the effectiveness of our proposed control method. The RMSE was used to evaluate the effectiveness of the control performance as follows:

(36) $$\begin{array}{l} RMSE=\sqrt{\frac{1}{n_{s}}\overset{n_{s}}{\underset{s=1}{\sum }}\left({\left(e_{\Theta }^{s}\right)}^{2}\right)}, \end{array}$$

where *n*_*s*_ is the number of samples.

**Table 3:** RMSE comparison for the quadcopter attitude control for the square wave commands.

**Controller**	**Computation time (ms)**	**Roll**	**Pitch**	**Yaw**
PID-MFNN	0.11444	0.01896	0.01902	0.07713
PID-CFNN	0.11093	0.02698	0.02177	0.07967

**Table 4:** RMSE comparison for the quadcopter attitude control for the sine wave commands.

**Controller**	**Computation time (ms)**	**Roll**	**Pitch**	**Yaw**
PID-MFNN	0.11444	0.0106	0.0106	0.1170
PID-CFNN	0.11093	0.0168	0.0154	0.1820

The parameter settings of the proposed PID-MFNN controller were chosen as *n*_*i*_ = 2, *n*_*j*_ = 3, and *n*_*k*_ = 2; the initial GMF for the fuzzy rules are given as *m*_*ij*1_ = [−0.6, 0, 0.6], *v*_*ij*1_ = [0.3, 03, 0.3], *m*_*ij*2_ = [−0.4, 0, 0.4], and *v*_*ij*2_ = [0.2, 02, 0.2]; the initial fuzzy weight is *w*_*klo*_ = 0.2; the learning rates were chosen as $\eta_{w}^{g}=\eta_{w}^{\alpha }=\eta_{m}^{\alpha }=\eta_{v}^{\alpha }=0.005$; the sampling time was 0.01 s. The same parameters were chosen for PID-CFNN, but only the number of layers was *n*_*k*_ = 1, and all GMFs were put in one layer.

The simulation results in Figures 8, 10 show that the proposed PID-MFNN controller has better performance than the PID-CFNN controller in terms of the rising time, settling time, and overshoot. The multilayer structure in our proposed controller, which contains a multilayer MF, provides it with a better scope regarding errors and disturbances compared with the single MF structure in PID-CFNN. The proposed PID-MFNN had a little longer computation time than the PID-CFNN due to the processing time of the multilayer structure. However, this did not affect the control performance, and the proposed PID-MFNN controller could achieve a smaller RMSE in both simulation cases. Apply some methods such as latent analysis in Wu et al. (2019) and Wu et al. (2020) to reduce the computational cost for the proposed multilayer fuzzy neural network will be our future work.

***Remark 1*.** The conventional PID usually hard to achieve high control performance for controlling non-linear systems due to its parameters are usually tuned for local points based on a linearization method (Mohan and Sinha, 2008; Cetin and Iplikci, 2015). Therefore, it is not suitable for controlling highly complex and non-linear systems, such as quadcopters. However, in our proposed PID-MFNN method, we designed adaptation laws for updating the MFNN network parameters online, so the output of the MFNN network can auto-tune the PID gains. Therefore, our proposed PID-MFNN is suitable for quadcopters as well as other non-linear control systems.

***Remark 2*.** By separating the member functions into a multilayer structure, the proposed method can better cover the changes in inputs. Moreover, with the proposed multilayer structure, the learning ability and flexibility of the network can be further improved.

***Remark 3*.** The difficulties and limits in this study are described as follows. When applying our method in an experiment with real quadcopters, suitable initial parameters needed to be chosen. Otherwise, control could be lost. In our study, the simulation results were obtained using a Gazebo robot simulator, and it was easy to choose these parameters using the trial-and-error method. In our future work, we plan to apply some optimal algorithms to realize suitable initial parameters.

## Conclusion

In this study, auto-tuning PID parameters using MFNN were successfully designed for controlling the attitude of quadcopters. The proposed method provided an effective method for obtaining suitable gains for PID controllers without the need for a mathematical system model or complicated calculations. In addition, a new multilayer structure was provided to improve the learning ability and flexibility of the used FNN. The parameters of the proposed network could be updated online using adaptation laws. Finally, the effectiveness of the proposed method was illustrated through the results of the conducted numerical simulations of quadcopter attitude control using a Gazebo robotics simulator and ROS. Our designed controller can also apply to a real quadcopter, and in our future work, we plan to apply some optimal algorithms to achieve suitable initial parameters.

## Data Availability Statement

The original contributions presented in the study are included in the article/supplementary material, further inquiries can be directed to the corresponding author/s.

## Author Contributions

All authors listed have made a substantial, direct and intellectual contribution to the work, and approved it for publication.

## Conflict of Interest

The authors declare that the research was conducted in the absence of any commercial or financial relationships that could be construed as a potential conflict of interest.
